# Supplementary material for: An ELISA-based method for Galleria mellonella apolipophorin-III quantification
Source: PeerJ. 2024 Mar 15;12:e17117. doi: 10.7717/peerj.17117 (PMC10946395; doi:10.7717/peerj.17117)
Supplement: Supplemental Information 1 [file peerj-12-17117-s001.pdf]

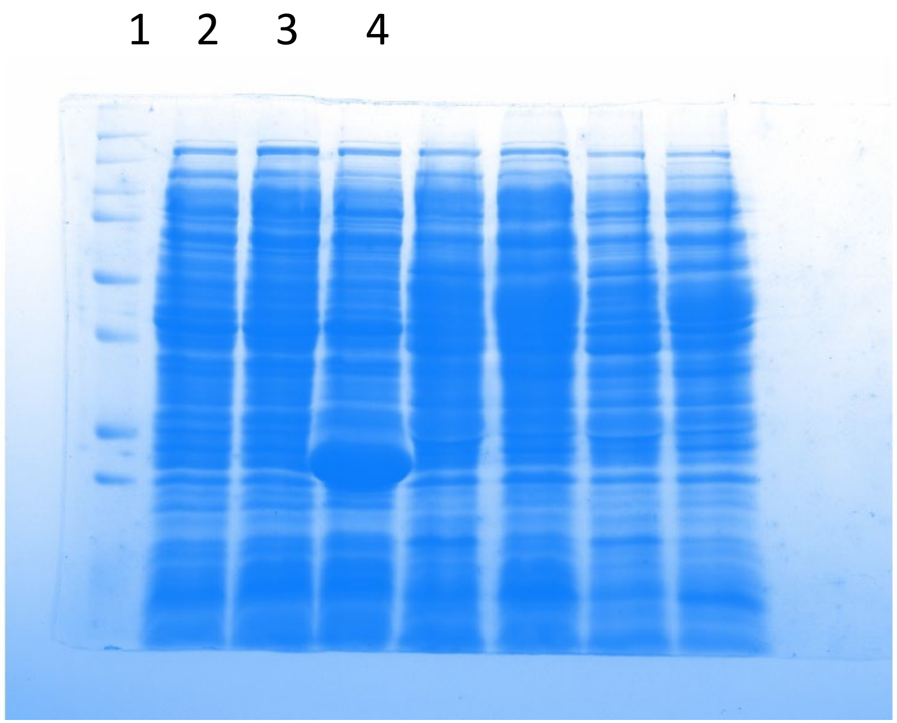

Original image of Figure 1, panel (A).
Line 1: molecular protein marker
Line 2: homogenate of non-transformed E. coli BL21.
Line 3: homogenate of E. coli BL21 transformed with empty vector.
Line 4: homogenate of E. coli BL21 transformed with pCold-ApoLpIII.
The gel was Coomassie blue-stained

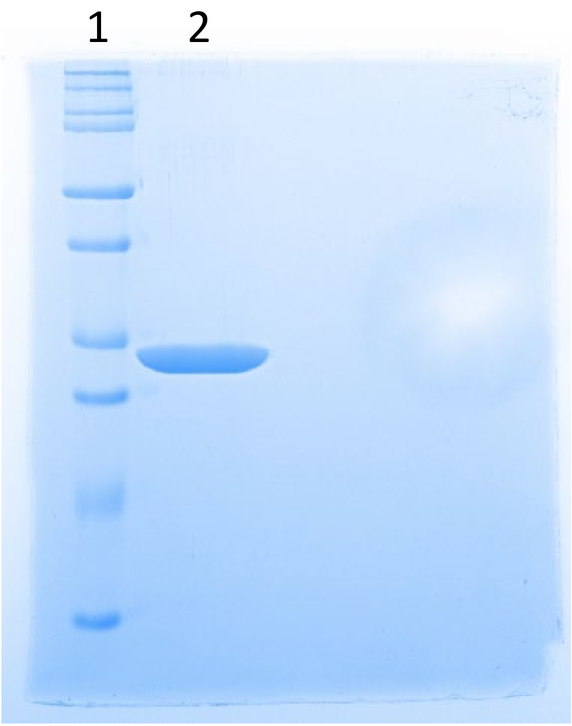

Original image of Figure 1, panel (B).
Line 1: molecular protein marker
Line 2: rApoLp-III purified and Coomassie blue-stained.

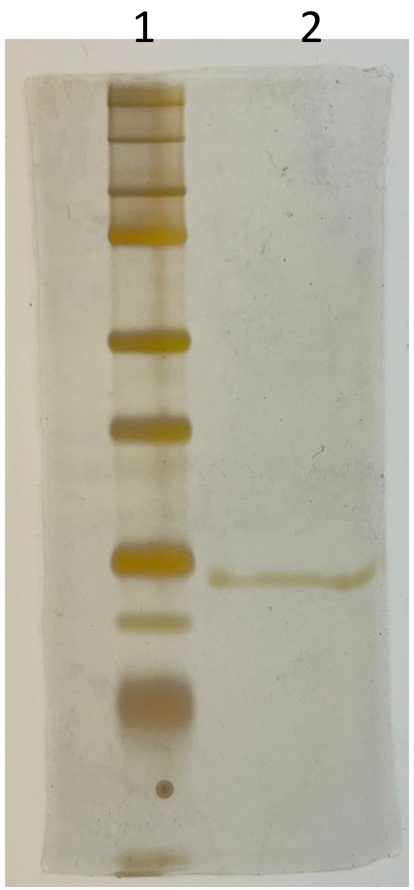

Original image of Figure 1, panel (C).
Line 1: molecular protein marker
Line 2: rApoLp-III purified and silver-stained.

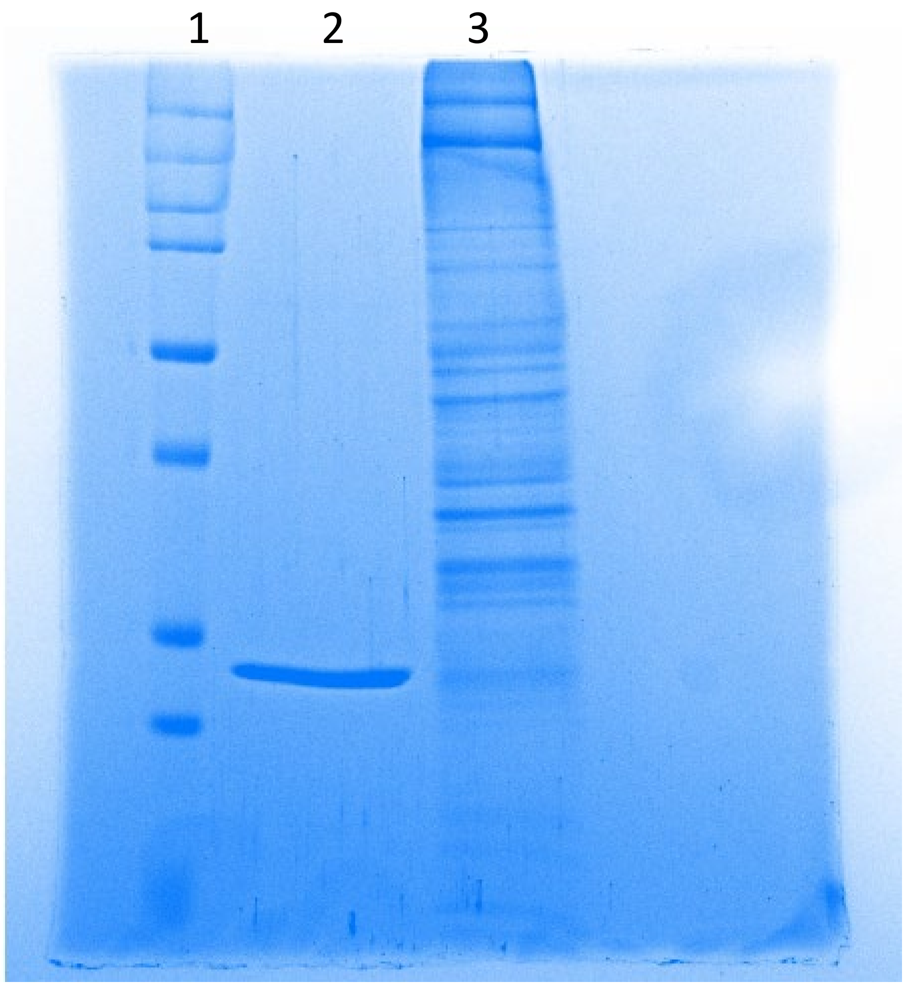

Original image of Figure 2, panel (A).

Line 1: molecular protein marker

Line 2: : rApoLp-III purified.

Line 3: *G. mellonella* moth's homogenate

The gel was Coomassie blue-stained

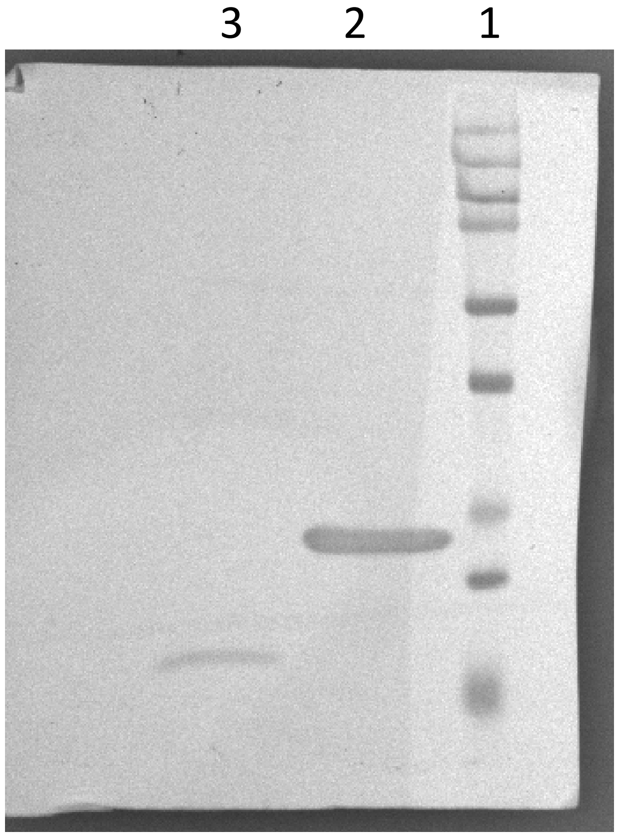

Original image of Figure 2, panel (B)

Line 1: molecular Protein marker

Line 2: rApoLp-III purified

Line 3: *G. mellonella* moth's homogenate

The membrane was incubate

[illegible]

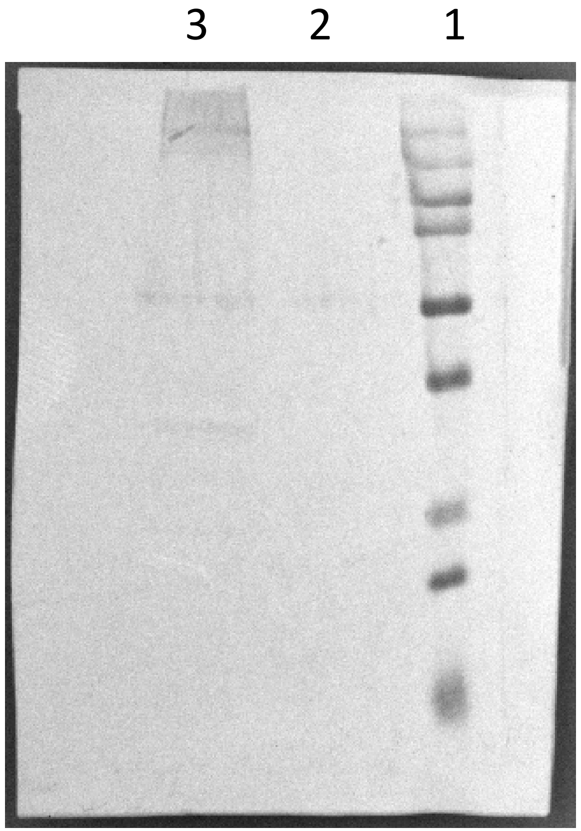

Original image of Figure 2, panel (C)

Line 1: molecular Protein marker

Line 2: rApoLp-III purified

Line 3*: G. mellonella* moth's homogenate

The membrane was incubated with pre-immune serum and

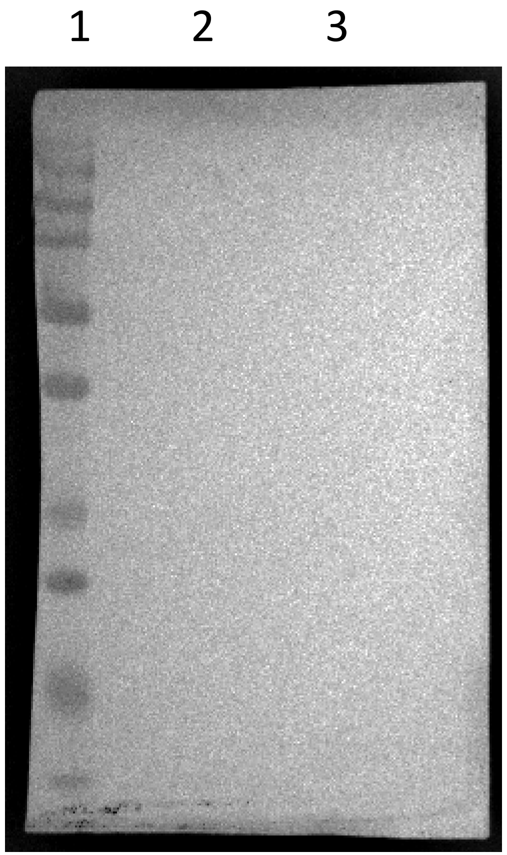

Original image of Figure 2, panel (D)

Line 1: molecular Protein marker

Line 2: rApoLp-III purified

Line 3*: G. mellonella* moth's homogenate

The membrane was incubated only with second antibody
